# Quantification of the Role of Teupol^®^ 25P and Graminex^®^ G96 Compared to Hexanic Extract of *Serenoa repens* in Patients Affected by Lower Urinary Tract Symptoms During Treatment with Silodosin

**DOI:** 10.3390/medicina61071225

**Published:** 2025-07-06

**Authors:** Yazan Al Salhi, Damiano Graziani, Andrea Fuschi, Fabio Maria Valenzi, Manfredi Bruno Sequi, Paolo Pietro Suraci, Alice Antonioni, Onofrio Antonio Rera, Cosimo De Nunzio, Riccardo Lombardo, Paolo Benanti, Giuseppe Candita, Eleonora Rosato, Filippo Gianfrancesco, Giorgio Martino, Giovanni Di Gregorio, Luca Erra, Giorgio Bozzini, Antonio Carbone, Antonio Luigi Pastore

**Affiliations:** 1 Urology Unit, Department of Medico-Surgical Sciences and Biotechnologies, Faculty of Pharmacy and Medicine, Sapienza University of Rome, Via Franco Faggiana 1668, 04100 Latina, Italy; 2 Department of Urology, Sant’Andrea Hospital, Sapienza University of Rome, Via di Grottarossa 1035/1039, 00189 Rome, Italy; 3 Unit of Urology, Fondazione PTV Policlinico Tor Vergata, Viale Oxford 81, 00133 Rome, Italy; 4 Department of Urology, ASST Lariana-Sant’Anna Hospital, Via Ravona, 20, San Fermo della Battaglia (CO), 22042 Como, Italy

**Keywords:** lower urinary tract symptoms, LUTS, benign prostatic hyperplasia (BPH), Xipag^®^, teupolioside, Graminex^®^, *Serenoa repens*, silodosin, phytotherapy, PSA, quality of life (QoL)

## Abstract

*Background and Objectives*: While α1-blockers like silodosin are the mainstay for treating lower urinary tract symptoms (LUTS) due to benign prostatic hyperplasia (BPH), combination therapy with phytotherapeutics may provide enhanced symptom control. Xipag^®^ is a novel formulation containing Graminex^®^ G96 (pollen extract) and Teupol^®^ 25P (teupolioside), offering anti-inflammatory and antiandrogenic effects. This study aimed to evaluate the efficacy of Xipag^®^ versus hexanic extract of *Serenoa repens* (HESr), both in combination with silodosin, in patients with LUTS/BPH. *Materials and Methods*: We conducted a single-center, prospective, observational, comparative study involving male patients with moderate-to-severe LUTSs undergoing treatment with silodosin. Patients were allocated to receive either Xipag^®^ or HESr in addition to silodosin, with follow-up every 3 months for 12 months. Primary outcomes included changes in symptom scores such as IPSS, QoL, and functional improvements such as peak urinary flow rate (Qmax). Multivariable regression analyses were used to assess predictors of the response. *Results*: Patients receiving Xipag^®^ showed significantly greater improvements in Qmax at all follow-up points (*p* < 0.05), with earlier and more sustained benefits compared to the HESr group. QoL index scores and PSA levels were also significantly better in the Xipag^®^ group starting from month six onward. IPSS scores improved in both groups but were significantly lower in the Xipag^®^ group only at 12 months (*p* = 0.04). No differences in erectile function (IIEF-5) or adverse events were observed. *Conclusions*: Xipag^®^ in combination with silodosin provides superior improvement in urinary flow, symptom-related QoL, and PSA reduction compared to HESr plus silodosin, with a favorable safety profile. These findings support the use of multi-target nutraceuticals like Xipag^®^ as a valuable adjunct in the management of LUTS/BPH. Larger randomized trials are warranted to confirm these results and explore underlying mechanisms.

## 1. Introduction

Historical records dating back to 3000 B.C. in ancient Egypt and China show the extensive use of herbal medicine. Despite the advent of purified pharmaceutical compounds in the 19th century, plant and herbal-based treatments still play an important role in modern medicine, particularly in urology [1]. Among the multitude of plant-based phytotherapeutic agents, hexanic extract of *Serenoa repens* (HESr) stands out as one of the herbal preparations endorsed by the European Medicines Agency’s (EMA) Committee on Herbal Medicinal Products (HMPC) for the treatment of lower urinary tract symptoms (LUTS) secondary to benign prostatic hyperplasia (BPH) [2]. Several studies have shown the efficacy and safety of HESr, demonstrating the ability to improve peak urinary flow rate (Qmax) and reduce symptoms such as nocturia [3,4,5]. The current EAU guidelines on the management of non-neurogenic male LUTS recommend managing patients with moderate to severe LUTS through the use of α1blockers (A1Bs, such as silodosin), due to the rapid onset of action, good efficacy, and low rate of adverse events [6]; however, the AUA guidelines are more conservative and currently state that the evidence supporting phytotherapeutic use remains insufficient for widespread recommendation, highlighting the need for further high-quality studies [7]. These compounds show their effectiveness in reducing International Prostatic Symptoms Scores (IPSS) and increasing the peak urinary flow rate (Qmax) when compared to a placebo [6]. In clinical practice, A1Bs are often prescribed in combination with HESr since a considerable proportion of patients experience persistent LUTS [8] and are not willing to undergo combination treatment with 5α-reductase inhibitors (5ARis) due to possible sexual dysfunction [6,9]. Combination therapies yield greater clinical improvement than monotherapy alone [10], due to the added anti-inflammatory, antiandrogenic, and antiproliferative properties of HESr [11,12,13]. To date, phytotherapeutic options are available as either monotherapy (e.g., HESr alone) or as combination preparations containing multiple active herbal compounds [6]. One emerging combination compound aimed to treat LUTS secondary to BPH is Xipag^®^ (IDI Integratori Dietetici Italiani S.r.l., Aci Bonaccorsi (CT), Italy), a fixed combination of a rye pollen extract with anti-inflammatory properties (Graminex^®^ G96; 500 mg) plus teupolioside (Teupol 25P^®^; 15 mg, an extract from immortalized cell cultures of Ajuga reptans), which has antiandrogenic properties. Early clinical data regarding Xipag^®^ suggest that it could improve LUTS secondary to BPH, without producing notable adverse reactions [14]. In this context, our study aims to determine whether the addition of Xipag^®^ to patients in ongoing treatment with silodosin yields better outcomes than the established combination of silodosin and HESr.

The choice of HESr as the comparator was guided by its widespread use in clinical practice and its inclusion in the EAU guidelines for the management of non-neurogenic male LUTS [6]. HESr is among the few phytotherapeutics with evidence from randomized controlled trials and meta-analyses [4,5]. Furthermore, HESr is also the most commonly prescribed plant-based agent in Europe for BPH management, especially in patients who decline 5ARis due to concerns about sexual dysfunction [9]. This pragmatic head-to-head comparison aligns with guideline flexibility and reflects typical physician decision-making patterns.

## 2. Materials and Methods

### 2.1. Study Design

We conducted a single-center, prospective, observational comparative study between March 2024 and March 2025 at the Department of Urology, Sapienza University of Rome, Polo Pontino, ICOT (Latina, Italy). Patients were assigned to treatment with either Xipag^®^ or HESr at the discretion of the treating physician, based on routine clinical judgment. The study adhered to the principles of the Declaration of Helsinki [15] and was approved by the local ethics committee. All participants provided written informed consent prior to enrollment.

### 2.2. Study Population

In March 2024, male patients with moderate to severe LUTS referred to our center as outpatients were enrolled in our study. Enrolment criteria were represented by the following: patients aged between 50 and 75 y.o., in treatment with silodosin 8 mg/day for at least 3 months prior to enrolment, and Qmax between 6 and 12 mL/s, with serum PSA levels ≤ 4 ng/mL or ≤ 10 ng/mL if corroborated by a negative prostate biopsy and or negative multiparametric prostate MRI (mpMRI). Patients were excluded from enrolment if there was suspicion of prostate cancer (e.g., positive digital rectal examination, imaging, or PSA level), history of prostate surgery, prostate cancer or pelvic radiation therapy, presence of neurogenic LUTS, urinary tract infection, and a post-void residual (PVR) ≥100 mL; known hypersensitivity to pollen extracts, teupolioside, or *Serenoa repens*; and use of 5ARis, phytotherapy, or anticholinergics within 3 months before enrolment. Each patient included was preliminary investigated at baseline (T0, all in treatment with silodosin 8 mg/day) assessing the following parameters: Qmax, PVR, IPSS, quality of life (QoL) index score, International Index of Erectile Function-5 (IIEF-5) score, total serum PSA, prostatic volume and middle lobe presence, evaluated through suprapubic ultrasound (SUS), or mpMRI, history of diabetes mellitus (DM), metabolic syndrome (MetS), smoking habit and body mass index (BMI), and whether they were in treatment with silodosin 8 mg/day for more than 1 year.

### 2.3. Treatment Allocation and Follow-Up Schedule

After baseline evaluation (T0), patients were divided into Group A, where patients received Xipag^®^ 1 tablet/day (500 mg of Graminex^®^ G96 pollen extract and 15 mg Teupol^®^ 25P) in addition to silodosin 8 mg, or Group B, where patients received 320 mg/day of HESr in addition to silodosin. The treatment duration was 12 months in both groups, with follow-up visits scheduled every 3 months (T1–T4). At each visit, the patients were administered the IPSS, IIEF-5, and QOL index questionnaires. We also evaluated Qmax, PVR, and serum PSA levels at each time point, while prostate volume was measured again at T4 through mpMRI. Adherence to therapy was verified through patient diaries and pill counts at each visit, and any adverse events were recorded. Treatment allocation was based on the attending physician’s clinical judgment, following standard care protocols. This pragmatic, real-world design reflects typical clinical decision-making, but introduces a potential risk of selection bias. No randomization or blinding was performed, and the possibility of unmeasured confounding, such as differences in patient expectations, health awareness, or physician prescribing preferences, cannot be excluded.

### 2.4. Statistical Analysis

Statistical analyses were conducted using SPSS Statistics version 29.0 (IBM Corp., Armonk, NY, USA). Continuous variables were presented as mean and standard deviation (SD) and compared between groups using the Student’s *t*-test or the Mann–Whitney U test. Categorical variables were expressed as absolute values and percentages and compared using the chi-square or Fisher’s exact test, as appropriate. A *p*-value < 0.05 was considered statistically significant. To evaluate clinical outcomes over time, comparisons of functional parameters between treatment groups were performed at each follow-up point (T1–T4). Multivariable linear regression analyses were conducted to identify independent predictors for statistically significant variables at T1, T2, T3, and T4. Each model was adjusted for relevant baseline covariates. Results are reported as beta coefficients (β) with 95% confidence intervals (CI) and corresponding *p*-values. Multivariable linear regression models included clinically relevant covariates determined a priori. Collinearity was assessed through variance inflation factors, and no significant multicollinearity was detected.

## 3. Results

### 3.1. Baseline Characteristics

The analysis included 33 patients in the silodosin plus Xipag^®^ group (Group A) and 42 patients in the silodosin plus HESr group (Group B). At baseline, the two treatment groups were balanced across all evaluated parameters. The mean age was 64.45 ± 5.43 years in Group A and 63.94 ± 5.61 years in Group B (*p* = 0.70). BMI was comparable between groups (25.94 ± 2.90 vs. 25.23 ± 2.37 kg/m^2^, *p* = 0.27, in groups A and B, respectively). Similarly, no significant differences were observed in Qmax (10.65 ± 2.29 vs. 10.59 ± 2.20 mL/s, *p* = 0.94), PVR volume (47.36 ± 19.39 vs. 44.41 ± 22.01 mL, *p* = 0.56, in groups A and B, respectively), symptom severity (IPSS: 17.27 ± 3.54 vs. 16.94 ± 3.95, *p* = 0.71), or quality of life (QoL index: 4.27 ± 0.58 vs. 4.14 ± 0.59, *p* = 0.37). Baseline PSA values (2.96 ± 1.86 vs. 2.91 ± 1.92 ng/mL, *p* = 0.89) and prostate volume (44.10 ± 24.49 vs. 44.47 ± 25.73 mL, *p* = 0.94) were also similar. The prevalence of MetS, DM, smoking habit, and presence of a median lobe did not differ significantly between groups. Baseline characteristics are summarized in Table 1.

### 3.2. Follow-Up Outcomes

**Figure 1:**
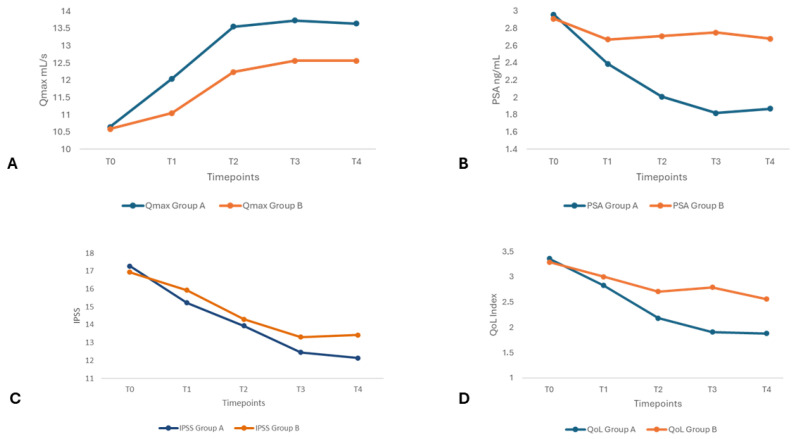
Longitudinal changes in key clinical outcomes over 12 months in Group A vs. Group B. (**A**) Qmax, mL/s over 12 months; (**B**) PSA, ng/mL over 12 months; (**C**) IPSS scores over 12 months; and (**D**) QoL index scores over 12 months. Abbreviations: Qmax, peak urinary flow rate; PSA, prostate-specific antigen; QoL, quality of life; and IPSS, International Prostate Symptom Score.

At T1 (3-month follow-up), patients in both groups showed early signs of clinical improvement, but differences were limited. Group A demonstrated a significantly higher Qmax than Group B (12.04 ± 1.50 vs. 11.05 ± 1.94 mL/s, *p* = 0.02). IPSS (*p* = 0.51), QoL index (*p* = 0.34), IIEF-5 (*p* = 0.80), PSA (*p* = 0.23), and PVR (*p* = 0.82) showed no statistically significant differences between groups. By six months (T2), differences between groups became more pronounced. Group A had a significantly higher Qmax (13.55 ± 2.00 vs. 12.24 ± 1.62 mL/s, *p* = 0.005) and lower QoL index scores (2.18 ± 0.77 vs. 2.71 ± 0.91, *p* = 0.01). IPSS and IIEF-5 scores were not found to be statistically significant (*p* = 0.43 and *p* = 0.83, respectively). PSA levels trended lower in Group A. However, this difference was not statistically significant (2.01 ± 1.23 vs. 2.71 ± 1.82 ng/mL, *p* = 0.07, in Groups A and B, respectively). PVR volume slightly favored Group A; however, the difference was not statistically significant (33.42 ± 16.19 vs. 39.26 ± 21.36 mL, *p* = 0.21, in Groups A and B, respectively). At the 9-month follow-up (T3), a statistically significant improvement was noted in Qmax (Group A 13.73 ± 1.92 vs. Group B 12.56 ± 1.37 mL/s, *p* = 0.006) and QoL (Group A 1.91 ± 0.84 vs. Group B 2.79 ± 0.81, *p* < 0.001). PSA levels were significantly lower in Group A (1.82 ± 1.34 vs. 2.75 ± 1.89 ng/mL, *p* = 0.026). No significant differences were observed in IIEF-5 (*p* = 0.79) or PVR (*p* = 0.22). IPSS scores at T3 approached significance (*p* = 0.08). At the final follow-up (T4, 12-month follow-up), Qmax remained significantly higher (13.64 ± 1.93 vs. 12.56 ± 1.42 mL/s, *p* = 0.012) and QoL scores remained better in Group A (1.88 ± 0.72 vs. 2.56 ± 0.89, *p* = 0.003). PSA levels were significantly lower in Group A (1.87 ± 1.41 vs. 2.68 ± 1.88 ng/mL, *p* = 0.03), supporting the potential biological activity of its antiandrogenic component. However, PSA reduction should not be interpreted as a definitive marker of clinical improvement, as it does not directly reflect symptom relief, prostate volume change, or mechanistic confirmation without supportive biomarker data. IPSS at the final follow-up improved in Group A (12.14 ± 2.37 vs. 13.43 ± 2.11, *p* = 0.04). Figure 1 graphically shows the changes over time of statistically significant variables.

IIEF-5 scores did not show significant variations across all follow-up timepoints and were not found to be statistically significant. PVR and prostate volume also did not differ significantly (PVR: *p* = 0.24; prostate volume: *p* = 0.92), although prostate volume slightly decreased in Group A compared to baseline (T0: 46.64 ± 23.89 mL vs. T4: 44.12 ± 20.09 mL). Table 2 summarizes the outcomes at all four follow-up points.

### 3.3. Multivariable Regression Analysis of Qmax, QoL, and PSA Outcomes

Multivariable regression analyses were conducted on Qmax, PSA, and QoL because these outcomes showed statistically significant differences between treatment groups in the unadjusted analyses. The aim was to assess whether treatment with Xipag^®^ remained an independent predictor of outcome after adjusting for baseline characteristics and potential confounders.

#### 3.3.1. Qmax

Xipag^®^ remained a significant independent predictor of increased Qmax at all timepoints (T1–T4 = β from 1.09–1.42, all *p* < 0.001). Silodosin use ≥1 year showed a negative effect at T1 and T2 (*p* = 0.014 and *p* = 0.020, respectively). However, it was not sustained at T3 and T4. Age showed a statistically significant negative association from T2 onward (T2: β = −0.05, *p* = 0.015, 95% CI: −0.091 to −0.01; T3: β = −0.04, *p* = 0.04, 95% CI: −0.087 to −0.003; and T4: β = −0.05, *p* = 0.027, 95% CI: −0.093 to −0.006), suggesting that older patients may have a less favorable improvement in urinary flow. BMI became a significant negative predictor at T4 (β = −0.097, *p* = 0.043, 95% CI: −0.189 to −0.005). Importantly, Qmax at T0 was a strong and consistent predictor of follow-up values at all timepoints (all *p* < 0.001), underscoring the predictive value of initial flow measurements. Smoking habit, MetS, and DM were not significantly associated with Qmax variations at any timepoint. The regression model is displayed in Table 3.

#### 3.3.2. QoL Index Score

Xipag^®^ treatment showed a significant reduction in QoL score at T2 (β = −0.54, *p* = 0.002, 95% CI: −0.87 to −0.2). This effect improved further at T3 (β = −0.90, *p* < 0.001, 95% CI: −1.25 to −0.54), and remained significant at T4 (β = −0.69, *p* < 0.001, 95% CI: −1.07 to −0.31). QoL score at T0 was a strong and consistent predictor of QoL at all follow-up points (all *p* < 0.001). Other covariates such as age, BMI, smoking, DM, and MetS did not show statistically significant associations at any follow-up point. The regression model is displayed in Table 4.

#### 3.3.3. PSA

At T1 Xipag^®^, the model showed a significant decrease in PSA (β = −0.28, *p* = 0.020, 95% CI: −0.51 to −0.04). The PSA-lowering effect of Xipag^®^ became evident at T2 (β = −0.84, *p* < 0.001, 95% CI: −1.01 to −0.59) and was statistically significant at T3 and T4, indicating a delayed but measurable impact (T3: β= −1.04, *p* < 0.001, 95% CI: −1.33 to −0.74 and T4: β= −1.08, *p* < 0.001, 95% CI: −1.26 to −0.61). PSA at T0 was the strongest predictor of serum PSA levels at every follow-up timepoint (*p* < 0.001). Interestingly BMI was found to be associated with an increase in PSA levels at T3 (β = 0.074, *p* = 0.023, 95% CI: 0.01 to 0.13) and T4 (β = 0.082, *p* = 0.034, 95% CI: 0.0095 to 0.19), suggesting a potential influence of body composition on PSA dynamics over time. The regression model is displayed in Table 5.

#### 3.3.4. IPSS

Baseline IPSS (T0) was a consistent and significant predictor of symptom scores at all follow-up timepoints (T1–T4, *p* ≤ 0.005). At 3 months, prior silodosin use ≥1 year was also associated with lower IPSS (β = −0.80, *p* = 0.042). Although Xipag^®^ showed a trend toward an association with reduced symptom scores at T2 and T3, the effect became statistically significant only at T4 (β = −0.49, *p* = 0.04). No significant associations were found for age, BMI, smoking status, MetS, or DM across any timepoint. The regression model is displayed in Table 6.

### 3.4. Adverse Reactions and Adherence

No adverse drug reaction or drug-drug interactions were recorded. Two patients in Group A and three in Group B did not adhere to treatment and were thus excluded from the analysis.

## 4. Discussion

Our 12-month real-world study comparing silodosin plus Xipag^®^ (a combination of pollen extract Graminex^®^ G96 and teupolioside) versus silodosin plus HESr showed that patients who received Xipag^®^ showed greater improvements in objective and patient-reported outcomes. In particular, patients receiving silodosin + Xipag^®^ showed a greater increase in Qmax and a more pronounced reduction in symptom-related QoL scores over the 12-month follow-up than patients treated with silodosin + HESr. PSA levels declined significantly in Group A by the end of the study. These findings suggest an additive benefit of the teupolioside/pollen combination in improving LUTS due to BPH. We observed a time-dependent onset of the phytotherapeutic benefits. The advantages in Group A became evident only after several months of therapy. Flow improvements in Group A began to exceed those in Group B by T1 significantly. Improvements in QoL scores emerged later, reaching significance around T2. The divergence in PSA levels between the two groups was more delayed. It is important to note that IPSS scores, while improving in both groups, did not differ significantly until T4, suggesting that treatment adherence is crucial. A significant reduction in PSA in Group A was observed at T3, even if the effect started by T2, whereas Group B showed no meaningful variation in PSA levels. This delayed onset aligns with the pharmacological action of phytotherapeutic agents, which typically require prolonged administration to exert maximal effects [6]. For instance, 5ARis require 6–12 months to achieve significant symptom and PSA improvements [16]; a nutraceutical with anti-androgenic and anti-inflammatory properties would also need time to induce measurable changes in prostate tissue [14,17]. Our findings are consistent with the existing literature that *Serenoa repens* monotherapy yields minimal symptom improvement compared to placebo after one year [18], and the benefits seem modest and have a slow onset. By contrast, the Xipag^®^ combination seems to accelerate and magnify the therapeutic response after the initial 3–6 month period, probably due to its multi-target mechanism of action. Teupolioside, a key component of Xipag^®^, is derived from Ajuga reptans cell cultures and exhibits anti-androgenic effects on prostatic cells. Teupolioside appears to reduce intraprostatic dihydrotestosterone (DHT) by depleting the NADPH coenzyme required for the conversion of testosterone into DHT, resulting in an antiandrogenic effect comparable to that of finasteride [14]. This may explain the PSA reduction in Group A: reduced DHT stimulation likely dampens glandular activity and PSA secretion [19]. However, this effect did not translate into a significant reduction in prostate volume, as would be expected with 5ARis. While Group A showed a significant reduction in PSA levels, we did not directly measure intraprostatic or serum DHT concentrations; thus, our findings regarding antiandrogenic activity remain inferential. In addition to teupolioside, the other main component is Graminex^®^ flower pollen extract, which contributes anti-inflammatory and anti-edema effects in the prostate [20]. Pollen extracts have a well-documented ability to alleviate prostatic inflammation: they reduce pro-inflammatory cytokine production (e.g., IL-1β, IL-6, TNF-α) and oxidative stress in the prostate, leading to resolution of glandular inflammation and stromal edema [21,22]. These anti-inflammatory actions can improve LUTSs by decreasing tissue swelling and outlet resistance, complementing silodosin’s smooth muscle relaxation [17]. Furthermore, Cai et al. described the role of pollen extracts in improving the QoL in patients affected by chronic prostatitis and chronic pelvic pain, and these results seem to be related to a reduction of IL-8 [23,24]. Moreover, Locatelli et al. found that Graminex^®^ reduces ROS production in immortalized prostate cells and MDA, NFκB mRNA, and PGE2 levels in rat prostate specimens [20]. In summary, Xipag^®^’s dual mechanisms likely represent the underlying reasons for the more robust improvements in Qmax and QoL we observed from T1– T4. Neither regimen adversely affected sexual function. IIEF-5 scores remained unchanged from baseline in both groups. This finding is clinically reassuring, especially given that combination therapy of αblockers (ABs) + 5ARis can have sexual side-effects such as erectile dysfunction and decreased libido. Nutraceuticals do not significantly alter systemic testosterone or the neural pathways of erection. Prior studies of pollen extracts in BPH have reported stable sexual function with a lack of sexual side-effects [25]. Additionally, no significant change in prostate volume was detected in either group after 12 months; while this might seem odd due to the antiandrogenic effects of Teupolioside, it is in line with existing evidence on phytotherapy. *Serenoa repens* is known not to significantly reduce prostate volume relative to a placebo in short-to-medium term trials [18]. Our results suggest that a 12-month period may be an insufficient timeframe to observe significant prostate volume reduction. Symptom improvement in BPH does not necessarily lead to prostatic volume reduction; reduced congestion, inflammation, and smooth muscle tone can enhance flow. The Qmax improvement in Group A verisimilarly indicates functional relief, a worthwhile trade-off given the symptom benefits without the side effects of more aggressive treatments. From a clinical standpoint, our findings suggest that adding the Xipag^®^ nutraceutical to an A1B may offer a valuable therapeutic strategy for men with moderate BPH symptoms, especially in cases where monotherapy with an A1B is suboptimal and the use of a 5ARi is undesired due to side effects. The Xipag^®^ combination was associated with better urinary flow and QoL outcomes than the widely used HESr, indicating that it may provide more effective adjunctive relief. It is also important to note that our interpretation of Xipag^®^’s antiandrogenic and anti-inflammatory effects is based on the prior literature rather than direct mechanistic evidence from this study. We did not measure intraprostatic DHT, serum testosterone, or inflammatory markers such as IL-6 or TNF-α. As such, the PSA reduction we observed, although statistically significant, should be considered a functional surrogate at best and does not confirm the proposed biological mechanism. Baseline prostatic inflammation was not assessed, further limiting interpretability.

Lastly, treatment adherence is crucial for achieving optimal outcomes in chronic conditions such as LUTSs/BPH. In our study, compliance was monitored through diaries and pill counts, though this method may be prone to bias. Two patients in Group A and three in Group B were excluded for poor adherence, indicating generally good tolerability. However, adherence rates were not formally quantified or correlated with outcomes, representing an important limitation. Future studies should incorporate validated adherence tools to better assess the real-world impact of nutraceutical therapies. The existing clinical evidence on Xipag^®^ remains limited to early-phase studies; further validation in larger controlled trials is needed.

## 5. Limitations

Several limitations of this study must be acknowledged. First, the sample size was relatively small, limiting the power to detect smaller differences between treatments and causing the possibility that some findings could be due to chance. Second, treatment allocation was non-randomized, potentially introducing selection bias. Although baseline characteristics were statistically balanced, the absence of randomization and blinding introduces the possibility of selection and performance bias, particularly for subjective endpoints such as IPSS and QoL. The observational nature of this study means that causality cannot be established as in a randomized controlled trial. Third, the follow-up duration of 12 months, while appropriate in assessing symptom changes, is relatively short for a chronic condition like BPH. Studies with a longer follow-up design are needed to determine if the benefits of Xipag^®^ persist, increase, or decrease over time, and whether it can impact long-term outcomes such as rates of acute urinary retention or need for BPH surgery. Finally, because we did not include a silodosin-only control arm, we cannot quantify the absolute benefit of either phytotherapeutic addition over AB monotherapy. Finally, although teupolioside is presumed to reduce DHT synthesis via NADPH depletion, we did not directly measure DHT levels. The PSA reduction observed in Group A may suggest an antiandrogenic effect, but this conclusion remains indirect. Future studies should incorporate DHT and inflammatory markers to validate the proposed mechanism and elucidate the biological significance of these findings. Despite these limitations, our study provides valuable preliminary evidence of the added benefit of a novel nutraceutical combination in the management of BPH.

## 6. Conclusions

In summary, the addition of Graminex^®^ G96 and teupolioside (Xipag^®^) to silodosin therapy may be associated with improvements in urinary flow, symptom-related QoL, and PSA levels over 12 months compared to the addition of HESr. The therapeutic benefits with Xipag^®^ appear after several months of continuous use, which is consistent with its anti-androgenic and anti-inflammatory mode of action. Teupolioside’s ability to diminish DHT production and Graminex^®^’s anti-inflammatory effects likely work in concert to achieve these outcomes. However, future trials should also perform biomarker analyses, including DHT and inflammatory cytokines, to validate the mechanistic underpinnings of Xipag^®^’s therapeutic effects. Both combination regimens were well-tolerated, with no negative impact on erectile function, and with no reported adverse reactions. These findings highlight a promising role for multi-component phytotherapy as an adjunct in treating LUTS/BPH. Given the observational nature of our study and its limited size, further larger randomized trials with longer follow-up are warranted to confirm these results and to better define which patient populations may derive the most benefits.

## Figures and Tables

**Table 1:** Baseline characteristics of the study population. Data are presented as mean (standard deviation) for continuous variables and percentages for categorical variables. No statistically significant differences were observed between groups at enrollment. BMI, body mass index; Qmax, peak urinary flow rate; PVR, post-void residual; IPSS, International Prostate Symptom Score; QoL, quality of life; IIEF-5, 5-item International Index of Erectile Function; DM, diabetes mellitus; MetS, metabolic syndrome; and SD, standard deviation.

Variable	Group A (33 Patients)	Group B (42 Patients)	*p*-Value
Age, years (SD)	64.45 (5.43)	63.94 (5.61)	0.7
BMI, kg/m2 (SD)	25.94 (2.90)	25.23 (2.37)	0.27
Qmax, mL/s (SD)	10.65 (2.29)	10.59 (2.20)	0.94
PSA, ng/mL (SD)	2.96 (1.86)	2.91 (1.92)	0.89
PVR, mL, (SD)	47.36 (19.39)	44.41 (22.01)	0.56
IPSS (SD)	17.27 (3.54)	16.94 (3.95)	0.71
QOL index (SD)	3.36 (1.08)	3.29 (0.87)	0.77
IIEF-5 (SD)	16.97 (2.60)	16.76 (2.24)	0.73
Prostate volume, mL (SD)	46.64 (23.89)	44.88 (27.35)	0.78
Silodosin ≥1 year, (%)	51.52	45.23	0.63
Smoking Habit (%)	27.27	26.19	0.93
DM (%)	21.21	19.04	0.76
MetS (%)	24.24	26.19	0.92
Median Lobe (%)	24.24	23.8	0.95

**Table 2:** Comparison of clinical and functional outcomes between Group A and B at each follow-up time point (T1: 3 months, T2: 6 months, T3: 9 months, and T4: 12 months). Data are presented as mean (standard deviation). Statistically significant differences (*p* < 0.05) are indicated in bold. Abbreviations: IPSS, International Prostate Symptom Score; QoL, quality of life index score; IIEF-5, 5-item International Index of Erectile Function; Qmax, peak urinary flow rate; PVR, post-void residual urine volume; PSA, prostate-specific antigen; and SD, standard deviation.

Variable	Group A	Group B	*p*-Value
**T1**			
IPSS (SD)	15.23 (2.62)	15.93 (2.63)	0.51
QOL (SD)	2.83 (1.29)	3.00 (0.82)	0.34
IIEF 5 (SD)	17.03 (2.63)	17.33 (2.07)	0.8
Qmax, mL/s (SD)	12.04 (1.50)	11.05 (1.94)	**0.02**
PVR, mL (SD)	39.94 (22.27)	41.12 (21.05)	0.82
PSA, ng/mL (SD)	2.39 (1.07)	2.67 (1.71)	0.23
**T2**			
IPSS (SD)	13.94 (2.40)	14.31 (2.24)	0.43
QOL (SD)	2.18 (0.77)	2.71 (0.91)	**0.01**
IIEF 5 (SD)	17.21 (2.54)	17.18 (2.07)	0.83
Qmax, mL/s (SD)	13.55 (2.00)	12.24 (1.62)	**0.005**
PVR, mL (SD)	33.42 (16.19)	39.26 (21.36)	0.21
PSA, ng/mL (SD)	2.01 (1.23)	2.71 (1.82)	0.07
**T3**			
IPSS (SD)	12.46 (2.31)	13.31 (2.18)	0.08
QOL (SD)	1.91 (0.84)	2.79 (0.81)	**<0.001**
IIEF 5 (SD)	16.98 (2.63)	17.29 (2.07)	0.79
Qmax, mL/s (SD)	13.73 (1.92)	12.56 (1.37)	**0.006**
PVR, mL (SD)	28.73 (11.21)	32.85 (15.78)	0.22
PSA, ng/mL (SD)	1.82 (1.34)	2.75 (1.89)	**0.026**
**T4**			
IPSS (SD)	12.14 (2.37)	13.43 (2.11)	**0.04**
QOL (SD)	1.88 (0.72)	2.56 (0.89)	**0.003**
IIEF 5 (SD)	17.13 (2.69)	17.27 (2.11)	0.84
Qmax, mL/s (SD)	13.64 (1.93)	12.56 (1.42)	**0.012**
PVR, mL (SD)	28.48 (10.97)	32.12 (14.27)	0.24
PSA, ng/mL (SD)	1.87 (1.41)	2.68 (1.88)	**0.03**
Prostate Volume, mL (SD)	44.12 (20.09)	44.65 (26.9)	0.92

**Table 3:** Multivariable linear regression models for Qmax. The models assess the independent effects of Xipag^®^ treatment and clinical covariates on Qmax over time, adjusting for baseline Qmax. At all timepoints, β coefficients represent the average change in Qmax (mL/s) for each predictor, with corresponding *p*-values and 95% confidence intervals. Qmax, peak urinary flow rate; BMI, body mass index; MetS, metabolic syndrome; DM, diabetes mellitus; CI, confidence interval; and β, coefficient.

Qmax T1					Qmax T2				
Predictor	β	*p*-Value	CI Lower	CI Upper	Predictor	β	*p*-Value	CI Lower	CI Upper
XIPAG^®^	1.09	<0.001	0.67	1.49	XIPAG^®^	1.42	<0.001	0.99	1.84
Silodosin ≥1 year	−0.55	0.014	−0.99	−0.124	Silodosin ≥1 year	−0.54	0.02	−0.99	−0.093
Age	−0.012	0.53	−0.05	0.026	Age	−0.05	0.015	−0.091	−0.01
BMI	−0.07	0.1	−0.15	0.012	BMI	−0.026	0.55	−0.11	0.06
Smoking habit	−0.46	0.19	−1.15	0.22	Smoking habit	0.38	0.3	−0.33	1.09
MetS	0.3	0.35	−0.32	0.93	MetS	0.39	0.25	−0.27	1.04
DM	−0.36	0.28	−1.03	0.29	DM	−0.63	0.075	−1.32	0.053
Qmax T0	0.61	<0.001	0.51	0.7	Qmax T0	0.65	<0.001	0.55	0.76
**Qmax T3**					**Qmax T4**				
**Predictor**	**β**	***p*-value**	**CI Lower**	**CI Upper**	**Predictor**	**β**	***p*-value**	**CI Lower**	**CI Upper**
XIPAG^®^	1.27	<0.001	0.77	1.78	XIPAG^®^	1.2	<0.001	0.74	1.66
Silodosin ≥1 year	−0.50	0.07	−1.03	0.032	Silodosin ≥1 year	−0.34	0.16	−0.82	0.14
Age	−0.04	0.04	−0.087	−0.003	Age	−0.05	0.027	−0.093	−0.006
BMI	−0.02	0.62	−0.12	0.076	BMI	−0.097	0.043	−0.189	−0.005
Smoking habit	0.33	0.44	−0.51	1.17	Smoking habit	−0.085	0.82	−0.85	0.68
MetS	0.36	0.36	−0.41	1.13	MetS	0.41	0.25	−0.28	1.12
DM	−0.76	0.07	−1.58	0.05	DM	−0.3	0.42	−1.04	0.43
Qmax T0	0.52	<0.001	0.4	0.65	Qmax T0	0.5765	<0.001	0.46	0.68

**Table 4:** Multivariable linear regression models for QoL index scores. Each model evaluates the independent association between Xipag^®^ treatment and QoL scores over time, adjusting for baseline QoL and other clinical covariates. At all timepoints, β coefficients represent each predictor’s average change in QoL Index scores, with corresponding *p*-values and 95% confidence intervals. QoL, quality of life; BMI, body mass index; MetS, metabolic syndrome; DM, diabetes mellitus; CI, confidence interval, and β, coefficient.

QoL Index T1					QoL Index T2				
Predictor	β	*p*-Value	CI Lower	CI Upper	Predictor	β	*p*-Value	CI Lower	CI Upper
XIPAG^®^	−0.1	0.55	−0.43	0.22	XIPAG^®^	−0.54	0.002	−0.87	−0.2
Silodosin ≥1 year	0.27	0.12	−0.068	0.62	Silodosin ≥1 year	−0.045	0.802	−0.39	0.3
Age	−0.005	0.74	−0.036	0.02	Age	−0.015	0.34	−0.04	0.016
BMI	0.017	0.61	−0.05	0.08	BMI	−0.035	0.31	−0.103	0.03
Smoking habit	−0.71	0.07	−1.26	0.15	Smoking habit	−0.13	0.63	−0.7	0.42
MetS	−0.38	0.14	−0.89	0.12	MetS	−0.51	0.067	−1.03	0.003
DM	0.35	0.2	−0.18	0.88	DM	−0.03	0.91	−0.57	0.51
QoL Index T0	0.92	<0.001	0.73	1.11	QoL Index T0	0.55	<0.001	0.36	0.74
**QoL index T3**					**QoL index T4**				
**Predictor**	**β**	***p*-value**	**CI Lower**	**CI Upper**	**Predictor**	**β**	***p*-value**	**CI Lower**	**CI Upper**
XIPAG^®^	−0.9	<0.001	−1.25	−0.54	XIPAG^®^	−0.69	<0.001	−1.07	−0.31
Silodosin ≥1 year	0.11	0.56	−0.26	0.48	Silodosin ≥1 year	0.091	0.65	−0.30	0.49
Age	−0.016	0.32	−0.049	0.016	Age	0.007	0.68	−0.028	0.042
BMI	−0.052	0.15	−0.12	0.019	BMI	−0.046	0.24	−0.12	0.03
Smoking habit	−0.36	0.23	−0.95	0.23	Smoking habit	−0.34	0.29	−0.98	0.29
MetS	−0.35	0.21	−0.89	0.19	MetS	−0.46	0.12	−1.04	0.12
DM	0.14	0.62	−0.43	0.71	DM	0.27	0.38	−0.34	0.89
QoL Index T0	0.47	<0.001	0.27	0.67	QoL Index T0	0.51	<0.001	0.29	0.73

**Table 5:** Multivariable linear regression models for total PSA levels. The analysis evaluates the independent impact of Xipag^®^ treatment and clinical covariates on PSA levels over time, adjusting for PSA at baseline. β coefficients represent the estimated change in PSA (ng/mL) for each predictor, with corresponding *p*-values and 95% confidence intervals. PSA; prostate-specific antigen; BMI, body mass index; MetS, metabolic syndrome; DM, diabetes mellitus; CI, confidence interval; and β, coefficient.

PSA T1					PSA T2				
Predictor	β	*p*-Value	CI Lower	CI Upper	Predictor	β	*p*-Value	CI Lower	CI Upper
Xipag^®^	−0.28	0.02	−0.51	−0.04	Xipag^®^	−0.84	<0.001	−1.01	−0.59
Silodosin ≥1 year	−0.05	0.69	−0.3	0.19	Silodosin ≥1 year	−0.107	0.43	−0.37	0.16
Age	−0.001	0.9	−0.023	0.02	Age	0.0095	0.42	−0.014	0.032
BMI	0.017	0.47	−0.03	0.06	BMI	0.056	0.033	0.0057	0.106
Smoking habit	0.22	0.27	−0.17	0.61	Smoking habit	0.24	0.26	−0.18	0.66
MetS	0.053	0.77	−0.309	0.41	MetS	0.007	0.97	−0.37	0.39
DM	−0.06	0.73	−0.44	0.31	DM	−0.028	0.89	−0.43	0.378
PSA T0	0.83	<0.001	0.75	0.91	PSA T0	0.9	<0.001	0.82	0.99
**PSA T3**					**PSA T4**				
**Predictor**	**β**	***p*** **-value**	**CI Lower**	**CI Upper**	**Predictor**	**β**	***p*** **-value**	**CI Lower**	**CI Upper**
Xipag^®^	−1.042	<0.001	−1.33	−0.74	Xipag^®^	−1.08	<0.001	−1.26	−0.61
Silodosin ≥1 year	−0.16	0.31	−0.47	0.15	Silodosin ≥1 year	−0.18	0.39	−0.51	0.23
Age	0.009	0.5	−0.01	0.03	Age	0.01	0.6	−0.022	0.044
BMI	0.074	0.023	0.01	0.13	BMI	0.082	0.034	0.0095	0.19
Smoking habit	0.305	0.23	−0.18	0.79	Smoking habit	0.33	0.31	−0.23	0.61
MetS	0.054	0.81	−0.4	0.5	MetS	0.059	0.79	−0.42	0.56
DM	0.043	0.86	−0.43	0.52	DM	0.051	0.88	−0.48	0.64
PSA T0	0.9	<0.001	0.80	0.99	PSA T0	0.93	<0.001	0.84	1.01

**Table 6:** Multivariable linear regression models for IPSS scores over time. The analysis assesses the independent effect of Xipag^®^ treatment and clinical covariates on symptom severity at 3, 6, 9, and 12 months. β coefficients represent the estimated change in IPSS for each predictor, with corresponding *p*-values and 95% confidence intervals. IPSS, International Prostate Symptom Score; BMI, body mass index; MetS, metabolic syndrome; DM, diabetes mellitus; CI, confidence interval; and β, coefficient.

IPSS T1					IPSS T2				
Predictor	β	*p*-Value	CI Lower	CI Upper	Predictor	β	*p*-Value	CI Lower	CI Upper
Xipag^®^	−0.3391	0.36	−1.08	0.405	Xipag^®^	−0.56	0.17	−1.3	0.25
Silodosin ≥1 year	−0.796	0.042	−1.56	−0.03	Silodosin ≥1 year	−0.40	0.43	−1.42	0.62
Age	−0.0205	0.54	−0.087	0.046	Age	−0.023	0.6	−0.11	0.06
BMI	−0.0604	0.42	−0.21	0.09	BMI	−0.082	0.42	−0.28	0.12
Smoking habit	−1.0834	0.089	−2.33	0.17	Smoking habit	−0.31	0.71	−1.98	1.36
MetS	0.49	0.38	−0.63	1.61	MetS	0.41	0.58	−1.09	1.91
DM	0.98	0.107	−0.21	2.18	DM	0.76	0.34	−0.84	2.36
IPSS T0	0.48	<0.001	0.36	0.600	IPSS T0	0.24	0.0028	0.08	0.41
**IPSS T3**					**IPSS T4**				
**Predictor**	**β**	***p*-value**	**CI Lower**	**CI Upper**	**Predictor**	**β**	***p*-value**	**CI Lower**	**CI Upper**
Xipag^®^	−0.22	0.12	−1.28	0.063	Xipag^®^	−0.49	0.04	−1.37	−0.07
Silodosin ≥1 year	−0.12	0.81	−1.22	0.97	Silodosin ≥1 year	−0.19	0.72	−1.30	0.91
Age	−0.03	0.43	−0.13	0.058	Age	−0.02	0.54	−0.12	0.067
BMI	−0.08	0.46	−0.3	0.13	BMI	−0.04	0.66	−0.26	0.17
Smoking habit	0.05	0.95	−1.74	1.85	Smoking habit	0.29	0.74	−1.51	2.1
MetS	0.76	0.34	−0.85	2.38	MetS	0.81	0.32	−0.81	2.43
DM	−0.21	0.8	−1.94	1.51	DM	−0.54	0.57	−2.27	1.19
IPSS T0	0.23	0.009	0.06	0.40	IPSS T0	0.21	0.015	0.0434	0.39

## Data Availability

Data are unavailable due to privacy or ethical restrictions imposed by our institution.

## Abbreviations

The following abbreviations are used in this manuscript:

A1B	α1-blocker
AB	α-blocker
BPH	benign prostatic hyperplasia
BMI	body mass index
CI	confidence interval
DM	diabetes mellitus
DHT	dihydrotestosterone
EMA	European Medicines Agency
HESr	hexanic extract of Serenoa repens
HMPC	Herbal Medicinal Products Committee
IIEF-5	5-item International Index of Erectile Function
IPSS	International Prostate Symptom Score
LPS	lipopolysaccharide
LUTS	lower urinary tract symptoms
MDA	malondialdehyde
MetS	metabolic syndrome
mpMRI	multiparametric magnetic resonance imaging
NADPH	nicotinamide adenine dinucleotide phosphate
NFκB	nuclear factor kappa B
PGE2	prostaglandin E2
PSA	prostate-specific antigen
PVR	post-void residual
QoL	quality of life
Qmax	peak urinary flow rate
ROS	reactive oxygen species
SD	standard deviation
SUS	suprapubic ultrasound
T0-T4	timepoints 0 to 4 (baseline to 12 months)
5ARi	5-alpha-reductase inhibitor
